# Phytochemical Constituents, Antioxidant and Antiproliferative Properties of a Liverwort, *Lepidozia borneensis* Stephani from Mount Kinabalu, Sabah, Malaysia

**DOI:** 10.1155/2015/936215

**Published:** 2015-11-11

**Authors:** Mohd Fadzelly Abu Bakar, Fifilyana Abdul Karim, Monica Suleiman, Azizul Isha, Asmah Rahmat

**Affiliations:** ^1^Faculty of Science, Technology and Human Development, Universiti Tun Hussein Onn Malaysia (UTHM), Parit Raja, 86400 Batu Pahat, Johor, Malaysia; ^2^Institute for Tropical Biology and Conservation, Universiti Malaysia Sabah, Jalan UMS, 88400 Kota Kinabalu, Sabah, Malaysia; ^3^Forest Research Centre, Sabah Forestry Department, P.O. Box 1407, 90715 Sandakan, Sabah, Malaysia; ^4^Laboratory of Natural Products, Institute of Bioscience, Universiti Putra Malaysia (UPM), 43400 Serdang, Selangor, Malaysia; ^5^Department of Nutrition and Dietetics, Faculty of Medicine and Health Science, Universiti Putra Malaysia (UPM), 43400 Serdang, Selangor, Malaysia

## Abstract

The study aimed to investigate the phytochemical contents, antioxidant and antiproliferative activity of 80% methanol extract of* Lepidozia borneensis*. The total phenolic and total flavonoid contents were analysed using Folin-Ciocalteu and aluminium chloride colorimetric methods. Antioxidant properties were evaluated by using FRAP, ABTS, and DPPH assays while the effects of* L. borneensis *on the proliferation of MCF-7 cell line were evaluated by using MTT assay. The results showed that the total phenolic and flavonoid contents were 12.42 ± 0.47 mg GAE/g and 9.36 ± 1.29 mg CE/g, respectively. The GC-MS analysis revealed the presence of at least 35 compounds. The extract was found to induce cytotoxicity against MCF-7 cell line with IC_50_ value of 47.33 ± 7.37 *µ*g/mL. Cell cycle analysis showed that the extract induced significant arrest at G_0_/G_1_ at 24 hours of treatment. After 72 hours of treatment, the proportion of cells in G_0_/G_1_ and G_2_-M phases had decreased significantly as compared to their control. Apoptosis occurred during the first 24 hours and significantly increased to 30.8% after 72 hours of treatment. No activation of caspase 3 was observed. These findings suggest that* L. borneensis *extract has the potential as natural antioxidant and anticancer agents.

## 1. Introduction

Approximately 14.1 million new cancer cases and 8.2 million deaths occurred in 2012 globally and the number of deaths caused by cancer is likely to increase to 13.1 million by 2030 [1, 2]. In Malaysia, cancer is ranked fourth among diseases that cause deaths [3]. According to the statistics by the Malaysian Ministry of Health, the annual mortality rate for cancer disease has reached more than 10% since 2006 [4]. More than 60% of currently used drugs for cancer treatment are natural based products [5]. The United States National Cancer Institute (NCI) has collected about 35,000 plants and screened 114,000 natural products extracts for anticancer studies [6].

Bryophytes have been commonly used as herbal medicine in China and India and among Native Americans since ancient times [7]. Bryophytes possess high amounts of terpenoids, phenolics, glycosides, fatty acids, and some rare aromatic compounds that might contribute to prevention of cancer and other chronic illnesses [8, 9]. Liverworts, in particular, have been widely use in traditional medicine. They contain lipophilic mono-, sesqui-, and diterpenoids, aromatic compounds (bibenzyls, bisbibenzyls, benzoates, cinnamates, long-chain alkyl phenols, naphthalenes, phthalides, and isocoumarins), and acetogenins which constitute the oil bodies that contribute to the biological activities [9]. There are more than 700 terpenoids and 220 aromatic compounds of liverworts that have been studied in terms of their biological activities [10].

Various compounds isolated from liverworts have high potential as chemotherapeutic agents. It has been reported that marchantin A, isoriccardin C, riccardin B, plagiochin E, and marchantin C isolated from* Reboulia hemisphaerica* were found to display cytotoxicity against EYFP-tubulin HeLa cells with IC_50_ values of 22.6 *μ*mol/L, 42.2 *μ*mol/L, 41.6 *μ*mol/L, 32.7 *μ*mol/L, and 23.2 *μ*mol/L, respectively [11]. Marchantin A which was isolated from* Marchantia emarginata* subsp.* tosana* was found to induce apoptosis in MCF-7 cells [12]. A macrocyclic bisbibenzyl, riccardin D, which was isolated from* Dumortiera hirsuta* inhibited the proliferation of human non-small-cell lung cancer (NSCLC) both* in vitro* and* in vivo* and does not cause toxicity in normal mammalian cells [13].

So far, the chemical compounds and biological activities of* Lepidozia borneensis* have not been extensively reported in literature. Thus, this study was carried out to determine the phytochemical constituents and investigate the antioxidants and antiproliferative effects of a liverwort,* L. borneensis*.

## 2. Materials and Methods

### 2.1. Plant Material and Sample Preparation

Plant sample was collected from Mount Kinabalu, Sabah, Malaysia. Voucher specimen was identified and deposited into BORNEENSIS Herbarium (BORH), Institute for Tropical Biology and Conservation, Universiti Malaysia Sabah, Kota Kinabalu, Sabah, Malaysia. The young green shoots of the bryophytes sample were cut using a pair of scissors, excluding the lower part which is normally brown in colour. Plant sample was carefully cleaned and washed by using distilled water to remove the contaminants. Sample was dried in the oven for two days at 40°C [14]. The dried sample was ground into fine powder using an electric blender.

### 2.2. Sample Extraction

Ground sample was extracted using the method from Velioglu et al. [15] with slight modification. A quantity of 0.1 g of sample was extracted using magnetic stirrer at 200 rpm for 2 h with 10 mL of 80% methanol at room temperature. The mixture was filtered through a filter paper. The extract was used for the determination of phytochemicals and antioxidant assessments. For antiproliferative analysis, the extract was evaporated using rotary evaporator and further freeze-dried using freeze-dryer.

### 2.3. Total Phenolic Content

Total phenolic content was determined using Folin-Ciocalteu reagent as adapted from Velioglu et al. [15] with modifications. An aliquot (100 *µ*L) of extract was mixed with 750 *µ*L of Folin-Ciocalteu reagent (previously diluted 10-fold with distilled water), vortexed, and allowed to stand at room temperature for 5 min. The mixture was then added to 750 *µ*L of sodium bicarbonate (60 g/L) solution. After 90 min at room temperature, absorbance was measured at 725 nm using spectrophotometer (Thermo Fisher Scientific 1510). Standards of gallic acid in the concentration ranging from 0 to 100 *µ*g/mL were run with the test samples. Results were expressed as mg gallic acid equivalent in 1 g of dried sample (mg GAE/g).

### 2.4. Total Flavonoid Content

The total flavonoid was measured according to method described by Zhishen et al. [16]. Briefly, 1 mL of extract was mixed with 4 mL of distilled water and 0.3 mL of 5% sodium nitrite solution. After 5 min, 0.6 mL of 10% aluminium chloride was added and allowed to stand for 6 min before 2 mL of 1 M sodium hydroxide was added to the mixture. The reaction of flavonoid with sodium nitrite and aluminium chloride produces coloured flavonoid-aluminium complex that was measured using spectrophotometer at 510 nm. Standards of catechin in the concentration ranging from 0 to 100 *µ*g/mL were run with the test samples. Results were expressed as mg catechin equivalent in 1 g of dried sample (mg CE/g).

### 2.5. Gas Chromatography Mass Spectroscopy

GC-MS analysis was done according to method described by Yong et al. [4]. Crude extract of plant sample was analysed with gas chromatography equipped with mass spectrometry (GC-MS-2010 Plus-Shimadzu). The column temperature was set to 50°C for 4 min before it was increased to 320°C at the rate of 7°C/min and was then held for 20 min. The injector temperature was set at 280°C (split mode with the ratio being adjusted to 20 : 1, injection volume = 0.1 *µ*L). The flow rate of the helium carrier gas was set to 1 mL/min with total run time of 60 min. Mass spectra were obtained from the range* m/z* 40 to 700 and the electron ionization at 70 eV. The chromatogram of the sample was identified by comparing their mass spectra with the library data and the GC retention time against known standards.

### 2.6. Antioxidant Assessment Parameters

#### 2.6.1. FRAP (Ferric Reducing/Antioxidant Power) Assay

This method was conducted according to Benzie and Strain [17] with slight modifications. The working FRAP reagent was produced by mixing 300 mM acetate buffer (pH 3.6), 10 mM 2,4,6-tripyridyl-s-triazine (TPTZ) solution, and 20 mM ferric chloride in a 10 : 1 : 1 ratio prior to use in water bath at 37°C. A total of 3 mL FRAP reagent was added to a test tube and a blank reading was taken at 593 nm using spectrophotometer. A total of 100 *µ*L of selected plant extracts and 300 *µ*L of distilled water were added to the test tube. After addition of the sample to the FRAP reagent, a second reading at 593 nm was performed after 4 min. The changes in absorbance after 4 min from initial blank reading were compared with standard curve, ferrous sulphate. Standards of known Fe (II) concentrations were run using several concentrations ranging from 200 to 1000 *µ*g/mL. A standard curve of FRAP values of each standard versus its concentration was plotted. The final result was expressed as mM ferric iron reduction to ferrous iron in 1 g of dry sample (mM/g).

#### 2.6.2. ABTS Decolourization Assay

The 2,2′-azinobis(3-ethylbenzothiazoline)-6-sulphonic acid or ABTS free radical decolourization assay was done according to Re et al. [18] with slight modifications. Working ABTS solution (7 mM) and 2.45 mM potassium persulfate were added to a beaker. The mixture was allowed to stand for 15 h in the dark at room temperature. The mixture was diluted with 80% methanol to obtain the absorbance of 0.7 ± 0.02 units at 734 nm. An aliquot of methanolic test solution of each sample (200 *µ*L) was added to 2 mL of ABTS free radical cation solution. It was then vortex vigorously. The absorbance was measured at 734 nm by using spectrophotometer. Standards of ascorbic acid in the concentration ranging from 0 to 80 *µ*g/mL were run with the test samples, from which a standard curve was plotted. The radical scavenging activity was expressed as mg ascorbic acid equivalent antioxidant capacity in 1 g of dry sample (mg AEAC/g).

#### 2.6.3. DPPH Free Radical Scavenging Assay

The antioxidant activity of the extract was measured by using 2,2-diphenyl-1-pycrylhydrazyl (DPPH) as a free radical model and a method adapted from Mensor et al. [19]. An aliquot (1 mL) of 0.3 mM methanol solution of DPPH was added to 2.5 mL sample or standards. The solutions were mixed vigorously and left to stand at room temperature for 30 min in the dark. The mixture was measured spectrophotometrically at 518 nm. The antioxidant activity (AA) was calculated as follows:

(1) AA%=100−Abs  sample−Abs  empty  sampleAbs  control×100,

where Abs is the following absorbance:

 Empty sample = 1 mL methanol + 2.5 mL extract,
 Control sample = 1 mL 0.3 mM DPPH + 2.5 mL methanol.

Absorbance of empty sample is equal to the absorbance of solvent added to extract without DPPH whereas absorbance of sample is equal to the absorbance of extract added to DPPH. The percentages of antioxidant activity of all samples were plotted. The final results were expressed as IC_50_ value (the concentration of sample producing 50% scavenging of the DPPH radical, *µ*g/mL).

### 2.7. Cytotoxicity Study

#### 2.7.1. Cell Culture

The hormone-dependent breast cancer (MCF-7) and mouse embryonic fibroblast (3T3) cell lines were obtained from American Type Culture Collection (ATCC, USA) and were grown in RPMI 1640 media with L-glutamine. Both cell lines were supplemented with 10% fetal bovine serum and 1% penicillin-streptomycin and incubated in CO_2_ incubator at 37°C.

#### 2.7.2. MTT Assay

A method described by Abu Bakar et al. [20] was referred for MTT assay. The cell viability was determined by staining with trypan blue. Growing cells were counted using haemocytometer and diluted in a culture medium to a density of 1 × 10^6^ cells/mL. From this cell suspension, 100 *µ*L was pipetted using multichannel pipette into 96-well microtitre plate and incubated at 37°C overnight. A serial dilution was added to each well of the plate starting from row A to row H from a concentration of 100 *µ*g/mL. The plate was incubated for another 72 h. After that, 20 *µ*L of MTT reagent was added to each well and incubated for four more hours. In order to dissolve and solubilize the coloured crystals, 100 *µ*L of solubilisation solution, dimethyl sulfoxide, was added to each well. The absorbance was finally read at 570 nm using ELISA reader (BMG LABTECH FLUOstar Omega) from which the cytotoxicity was determined by the following formula:

(2) $$\begin{array}{c} \text{Cytotoxicity}\left(\;\%\;\right)=\frac{\text{Optical density of sample}}{\text{Optical density of control}}\times 100. \end{array}$$

The inhibition concentration (IC_50_), concentration of extract that is able to inhibit cell proliferation by 50%, was calculated graphically for each cell proliferation curve.

#### 2.7.3. Cell Cycle Analysis

The cell cycle analysis was done according to method described by Abu Bakar et al. [20]. A total of 1 × 10^6^ cells were incubated and treated with the sample extracts at IC_50_ value for 24, 48, and 72 h. All adhering and floating cells were harvested and transferred to a sterile centrifuge tube. The cells were centrifuged using centrifuge machine at 4°C with 1,200 rpm for 10 min. Cells were washed using cold PBS and resuspended in 0.5 mL cold PBS. Ice-cold 70% ethanol was added to the cell suspension and incubated in −20°C for 2 h. The sample was then centrifuged and the ethanol was removed. The cells were washed twice using cold PBS before they were stained with 500 *µ*L of 10 *µ*g/mL propidium iodide in 100 *µ*g/mL of RNase for 30 min at room temperature in the dark. Cell cycle distribution was detected with flow cytometry. The results were analysed using Summit 4.3 software.

#### 2.7.4. Annexin V-FITC Early and Late Apoptosis

The apoptosis was determined using Annexin V-FITC Apoptosis Detection Kit (Catalog Number APOAF, Sigma). Cells at a concentration of 1 × 10^6^ cells were incubated and treated with the sample extracts at IC_50_ value for 24, 48, and 72 h. All adhering and floating cells were harvested, washed twice using PBS, and transferred to a sterile centrifuge tube. The cells were suspended in binding buffer (100 mM HEPES/NaOH, pH 7.5 containing 1.4 M NaCl and 25 mM CaCl_2_) at a concentration of 1 × 10^6^ cells per mL. A total of 500 *µ*L of the cells were transferred to a 5 mL culture tube. After that, 5 *µ*L of Annexin V-FITC conjugate and 10 *µ*L of propidium iodide were added to the cell suspension. The cells were incubated for 10 min at room temperature in the dark. The fluorescence of the cells was determined by flow cytometer. The results were analysed using Summit 4.3 software.

#### 2.7.5. Caspase-3 Activity Assay

The activity of caspase-3 was determined using Caspase-3 Colorimetric Assay Sample Kit (Catalog Number BF3100, R & D Systems). Treated and untreated cells with a total density of 1 × 10^6^ were harvested and transferred to a sterile test tube and lysed using the cell lysis buffer provided. The lysed cells (50 *µ*L) were then aliquoted into 96-well microtitre plate. A reaction buffer (50 *µ*L) containing 10 mM DTT was added. Substrates selective (DEVD-pNA) were added to appropriate wells and the plate was incubated at 37°C for 2 h. The absorbance was finally read at a wavelength of 405 nm using microplate reader. The absorbance of treated samples was compared with untreated control for the determination of any fold increase in caspase activity.

### 2.8. Statistical Analysis

All determinations were carried out in three replicates in three independent experiments. Results were expressed as mean ± standard deviation (SD). Flow cytometry data (cell cycle and annexin-V FITC) and caspase were analysed using Student's *t*-test (unpaired). *p*-value < 0.05 was regarded as significant.

## 3. Results

### 3.1. Total Phenolic Contents (TPC), Total Flavonoid Contents (TFC), and Phytochemical Profiling Using GC-MS

The results showed that the total phenolic and flavonoid contents of 80% methanol extract of* L. borneensis* were 12.42 ± 0.47 mg GAE/g and 9.36 ± 1.29 mg CE/g, respectively. The GC-MS analysis revealed the presence of at least 35 compounds for* L. borneensis* extract. The retention time taken by the bioactive compounds of the sample extract varied from 2.68 to 67.75. The list of bioactive compounds from the sample extract is tabulated in Table 1.

### 3.2. Antioxidant Properties

For FRAP assay, the extract of* L. borneensis* displayed high reducing ability with the value of 211.13 ± 0.89 mM Fe^2+^/g. The extract of* L. borneensis* also has the scavenging ability for ABTS assay with the value of 0.49 ± 0.00 mg AEAC/g whereas, for DPPH assay, the IC_50_ value was 216.67 ± 20.82 *µ*g/mL.

### 3.3. Anticancer Properties

**Figure 1:**
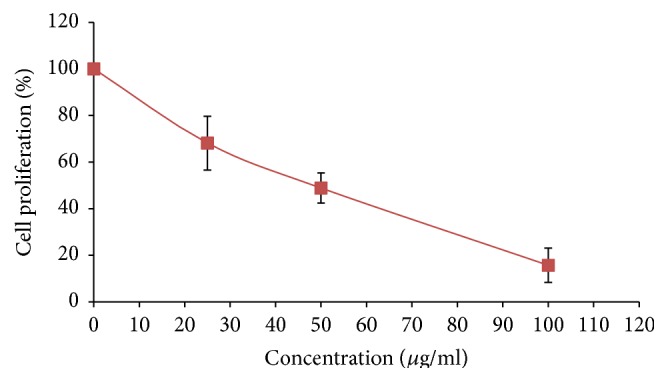
The effect of 80% methanol crude extract of* L. borneensis* on MCF-7 cell proliferation. Results are presented as mean ± standard deviation (*n* = 3). Cells (1 × 10^6^ cells/well) were treated with* L. borneensis* extract at different concentrations (0–100 *µ*g/mL) for 72 h. Cell proliferation was evaluated as the ability to reduce MTT to blue formazan crystals.

The results showed that the incubation of crude extract of* L. borneensis* with MCF-7 cell line inhibited cell proliferation with IC_50_ value of 47.33 ± 7.37 *µ*g/mL. The extract of* L. borneensis* did not display cytotoxic effect against 3T3 cell line. As illustrated in Figure 1, the incubation of* L. borneensis* extract with MCF-7 cells inhibited cell proliferation in a concentration dependent manner in the concentration ranging from 20 to 100 *µ*g/mL.

**Figure 2:**
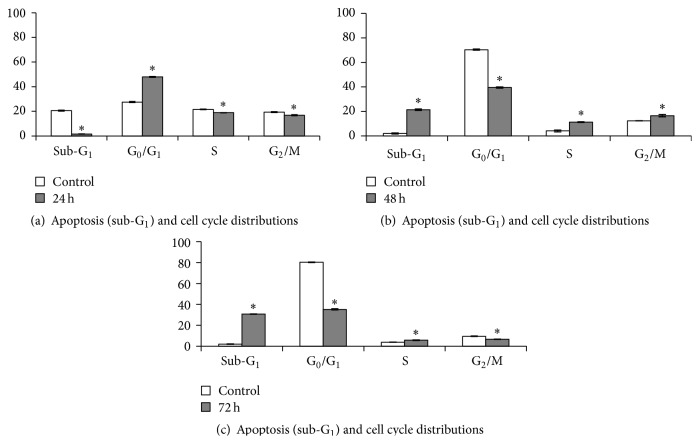
Cell cycle distribution of MCF-7 cancer cells treated with 80% methanol crude extract of* L. borneensis* at IC_50_ value. Values are expressed as mean ± standard deviation (*n* = 3). *∗* showed a significant difference (*p* < 0.05) relative to their respective control. The distribution of cells undergoing apoptosis and in various phases of the cell cycle was determined in MCF-7 cells treated with* L. borneensis* extract for 24 h (a), 48 h (b), and 72 h (c) in comparison to their respective control.

**Figure 3:**
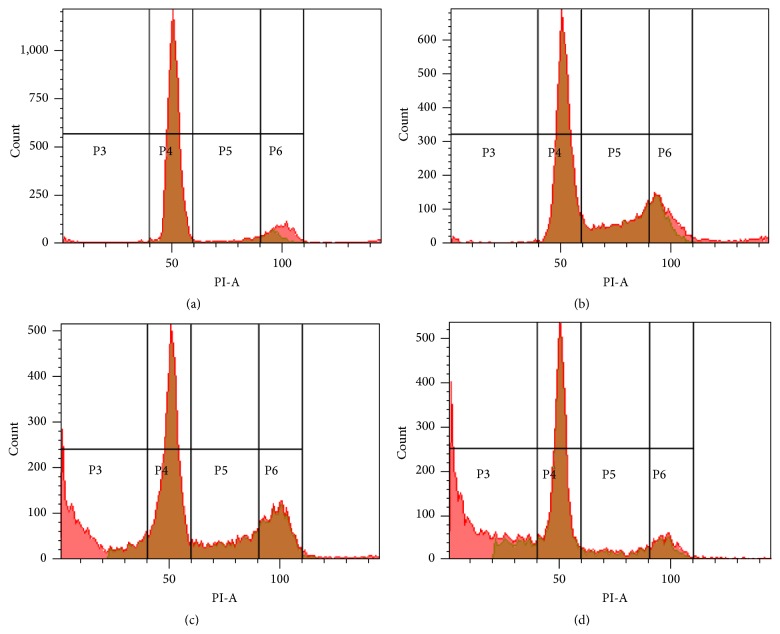
Flow cytometric scans of untreated MCF-7 (a) cancer cells and those treated with 80% methanol crude extract of* L. borneensis* at IC_50_ value for 24 h (b), 48 h (c), and 72 h (d). Sectors P3–P6 represent the cells in sub-G_1_, G_0_/G_1_, S, and G_2_-M phases, respectively.

Apoptosis and cell cycle distribution in MCF-7 cells were studied after exposure to* L. borneensis* extract at IC_50_ concentration for 24, 48, and 72 hours (Figure 3). There was a significant arrest at G_0_/G_1_ for MCF-7 cell lines at 24 hours of treatment with the crude extract of* L. borneensis*, whilst the number of cells in S and G_2_-M phases were reduced significantly (*p* < 0.05). After 48 hours of treatment, cells were higher in S and G_2_-M phases as compared to their respective control (*p* < 0.05). Apoptosis (sub-G_1_) occurred in the first 24 hours of the experiment. After 48 and 72 hours of treatment, the apoptosis phase had significantly increased to 21.4% and 30.8% (*p* < 0.05), respectively. In addition to this, the proportion of cells in G_0_/G_1_ and G_2_-M phases had decreased significantly as compared to their control after 72 hours of treatment (*p* < 0.05) (Figure 2).

**Figure 4:**
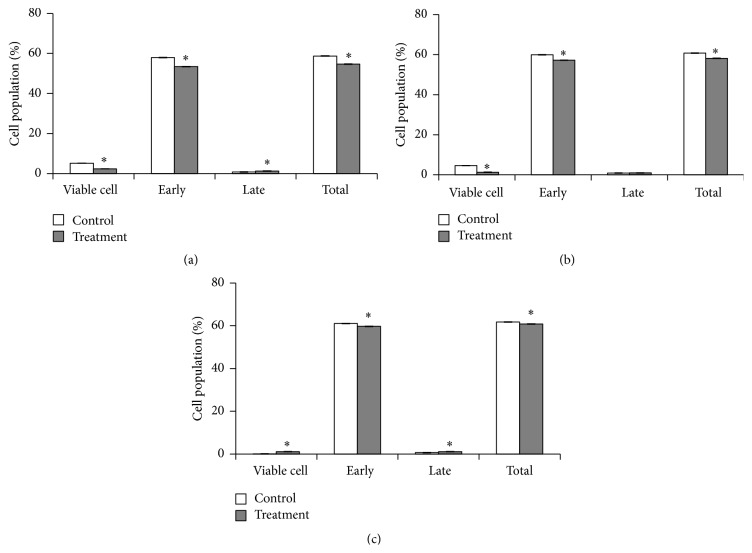
Apoptosis study of MCF-7 cells treated with 80% methanol crude extract of* L. borneensis* at IC_50_ value. Values are expressed as mean ± standard deviation (*n* = 3). *∗* showed a significant difference (*p* < 0.05) relative to their respective control. The distribution of cells undergoing early and late apoptosis together with those viable cells not in apoptosis and the total extent of apoptosis was determined in MCF-7 cells treated with* L. borneensis* extract for 24 h (a), 48 h (b), and 72 h (c) in comparison to their respective control, using Annexin-V FITC and propidium iodide flow cytometric analysis.

There was a significant difference in the proportion of cells that are undergoing early and late apoptosis detected in cells treated with the crude extract of* L. borneensis* if compared to control except for the late apoptosis for 48 hours of treatment (*p* < 0.05) (Figure 4). The proportion of cells in the live cell group decreased significantly from 2.4% for 24 hours treatment to 1.3% for 48 hours treatment with an increase in the proportion of total cells in early and late apoptosis from 54.7% for 24 hours treatment to 58.2% for 48 hours treatment. The total apoptosis increased up to 60.8% (comprised of early apoptosis: 59.7% and late apoptosis: 1.1%) at the end of 72 hours of treatment.

**Figure 5:**
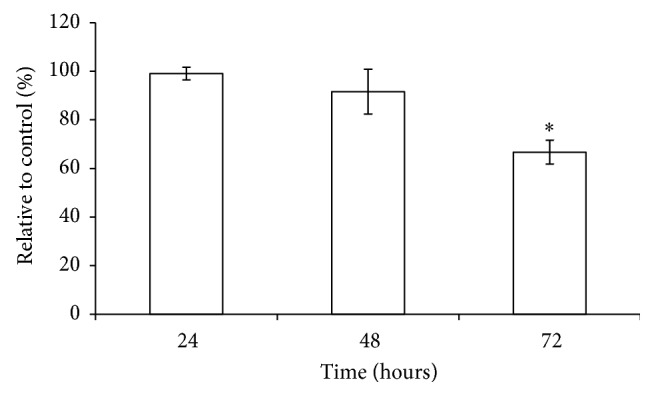
Caspase-3 activity by 80% methanol crude extract of* L. borneensis*. Values are expressed as mean ± standard deviation (*n* = 3).

The activity of caspase 3 was evaluated in MCF-7 cells to establish the cell death pathway induced by the extract of* L. borneensis*. However, there was no activation of caspase 3 observed. The time course of effect of crude extract of* L. borneensis* on the activity of the executioner caspase 3 is shown in Figure 5.

## 4. Discussion

This species has not been extensively studied in terms of its biological activities. This study reported the phytochemical profiling as well as the antioxidants and antiproliferative effects of* L. borneensis* extract against MCF-7 breast cancer cell line. The antioxidant potential of this species might be due to the phytochemical contents by acting as reducing agents or free radical scavengers and/or the synergistic effects by phenolics and flavonoids [21]. It has been reported that the biological activities of liverworts are due to the biological characteristics of terpenoids and aromatic compounds they possess [9]. The phytochemical contents of* L. borneensis* extract is comparable to other extracts of liverworts species such as methanol extract of* Plagiochila beddomei* (TPC = 19.3 mg/g, TFC = 16.9 mg/g) and ethanol extract of* Marchantia polymorpha* (TPC = 12.5 mg/g) [21, 22]. Similar to the extract of* L. borneensis*, both extracts also have the antioxidant properties.

The GC-MS analysis of the* L. borneensis* extract showed that 2-propyn-1-ol has the highest concentration among detected secondary metabolites. In contrast to the result of this study, a previous study showed that the major compound of* L. borneensis* is *β*-bazzanene, a common compound in* Bazzania* species which belong to the same family of* L. borneensis* (Lepidoziaceae) [23, 24]. A recent study reported that 1,3-dioxol-2-one,4,5-dimethyl which was also detected in* Hedychium coronarium in vitro* grown stem extract (2.28%, RT: 5.66) has antihypertensive property [25]. It was reported that the GC-qMS analysis revealed that 1,2-cyclopentanedione, a compound detected in* L. borneensis* extract, is also one of the volatile metabolites detected in* Carica papaya* [26]. Hexadecenoic acid was previously reported to have antioxidant property [27].

In the present study, certain compounds detected in the extract of* L. borneensis* were reported to have anticancer properties. Acetol or 2-propanone, 1-hydroxy- was reported to inhibit jack-bean urease activity with IC_50_ value of 2.9 mM [28]. Other than acetol, phytol which was present in the methanol extract of* Cassia italica* leaf (1.64%, RT: 18.47) has anticancer, antimicrobial, cancer preventive, and anti-inflammatory properties [27]. It has been suggested that the synergistic effects of the crude extract might reflect the anticancer property of sample extract [29]. Among flavonoids, apigenin, epigallocatechin gallate, delphinidin, and genistein have been stated as beneficial compounds in multistages of carcinogenesis [30]. A study conducted on* Marchantia linearis* showed that apigenin, quercetin, and luteolin are the major flavonoids identified in the extract and the result of the MTT assay showed that 50 *µ*g/mL of flavonoid induced cell proliferation as well as apoptosis in SW 480 colon cancer cell lines [31].

The transition of G_1_ to S phase requires a particular protein [32]. The treatment of* L. borneensis* extract to MCF-7 cells might alter the activity of this protein and caused cell cycle arrest in G_0_/G_1_ phase and a parallel reduction in G_2_ phase. An increased number of cells in sub-G_1_ indicated apoptosis event [33]. Cell cycle arrest caused by the treatment of* L. borneensis* extract might be associated with its phytochemical compounds. Saponins, triterpenes, sterols, and polyphenolic compounds were suggested to be responsible for the cytotoxicity against cancer cell lines [34]. In a previous study conducted on sasanquasaponin, a triterpenoid from* Camellia oleifera,* it was found to induce G_1_ cell cycle arrest and apoptosis of MCF-7 cells [35]. Curcumin, a natural polyphenol also induced G_1_ cell cycle arrest in MCF-7 cell line [36].

There was no induction of caspase 3 observed when MCF-7 cells were exposed to* L. borneensis* extract. There might be no involvement of caspase 8 or caspase 9 which will lead to the activation of caspase 3 [37, 38]. Apoptosis might occur using different pathways which omit the activation of caspases. It was suggested that either caspase-activated DNase is activated by another enzyme or a caspase-independent DNase is responsible for a DNA cleavage [39].

## 5. Conclusion

In conclusion, this study revealed that the extract of* L. borneensis* displayed antioxidant properties and was able to induce cell cycle arrest and apoptosis in hormone-dependent breast cancer cells. Thus, this species has the potential to be used as antioxidants and anticancer agents.

## Figures and Tables

**Table 1:** Secondary metabolites of crude extract of 80% methanol of *L. borneensis*.

Number	Retention time	Compound name	Concentration (%)
1	3.67	2-Propyn-1-ol	11.00
2	2.68	2-Propanone, 1-hydroxy-	9.93
3	58.00	2,2′-Ethylenediphenol	6.49
4	12.60	1,3-Dioxol-2-one,4,5-dimethyl	4.78
5	7.99	1,2-Cyclopentanedione	4.41
6	15.05	2-Propenoic acid, ethyl ester	4.22
7	49.73	Hexadecanoic acid, methyl ester	2.85
8	7.01	2-Propanone, 1,3-dihydroxy-	2.56
9	3.28	Glycerin	2.52
10	6.38	Propanoic acid, 2-methyl-, methyl ester	2.31
11	64.65	cis-anti-cis-Tricyclo[7.3.0.0(2,6)]dodecan-7-one	2.03
12	52.71	*α*-Patchoulene	1.82
13	21.28	2(3H)-Furanone, dihydro-5-pentyl-	1.76
14	30.45	Propanoic acid, 3-(acetyloxy)-2-(hydroxymethyl)-, ethyl ester, (+)-	1.59
15	67.20	Sandaracopimar-15-ene-6*β*,8*β*,11*α*-triol	1.40
16	21.63	3-Hepten-2-one, 4-methyl-	1.32
17	55.73	Phytol	1.30
18	59.19	6*β* Bicyclo[4.3.0]nonane, 5*β*-iodomethyl-1*β*-isopropenyl-4*α*,5*α*dimethyl-	1.25
19	51.04	n-Hexadecanoic acid	1.05
20	37.04	Spathulenol	0.98
21	26.60	1,4-Butandiol, 2,3-dimethoxy-	0.94
22	56.89	Elemol	0.82
23	60.25	2-[4-Methyl-6-(2,6,6-trimethylcyclohex-1-enyl)hexa-1,3,5-trienyl]cyclohex-1-en-1-carboxaldehyde	0.77
24	61.67	2-[4-Methyl-6-(2,6,6-trimethylcyclohex-1-enyl)hexa-1,3,5-trienyl]cyclohex-1-en-1-carboxaldehyde	0.73
25	55.53	9,12,15-Octadecatrienoic acid, methyl ester, (Z,Z,Z)-	0.73
26	62.94	9,19-Cycloergost-24(28)-en-3-ol, 4,14-dimethyl-, acetate, (3*β*,4*α*,5*α*)-	0.72
27	64.85	4,8,13-Cyclotetradecatriene-1,3-diol, 1,5,9-trimethyl-12-(1-methylethyl)-	0.67
28	55.31	9,12-Octadecadienoic acid (Z,Z)-, methyl ester	0.58
29	59.63	11*α*-Hydrozyresibufogenin	0.44
30	63.39	2-[4-Methyl-6-(2,6,6-trimethylcyclohex-1-enyl)hexa-1,3,5-trienyl]cyclohex-1-en-1-carboxaldehyde	0.37
31	65.07	Shyobunone	0.30
32	64.25	Spiro[2.5]octane, 5,5-dimethyl-4-(3-oxobutyl)-	0.26
33	50.08	Cycloheptane, 4-methylene-1-methyl-2-(2-methyl-1-propen-1-yl)-1-vinyl-	0.26
34	60.82	11*α*-Hydrozyresibufogenin	0.25
35	67.75	Ethyl iso-allocholate	0.24
